# Phosphorylation of NANOG by casein kinase I regulates embryonic stem cell self‐renewal

**DOI:** 10.1002/1873-3468.13969

**Published:** 2020-11-18

**Authors:** Nicholas P. Mullin, Joby Varghese, Douglas Colby, Julia M. Richardson, Greg M. Findlay, Ian Chambers

**Affiliations:** ^1^ Centre for Regenerative Medicine Institute for Stem Cell Research School of Biological Sciences University of Edinburgh UK; ^2^ Protein Phosphorylation and Ubiquitylation Unit James Black Centre School of Life Sciences Dundee UK; ^3^ Institute of Quantitative Biology, Biochemistry and Biotechnology Edinburgh UK

**Keywords:** casein kinase I, DNA binding, NANOG, phosphorylation, self‐renewal

## Abstract

The self‐renewal efficiency of mouse embryonic stem cells (ESCs) is determined by the concentration of the transcription factor NANOG. While NANOG binds thousands of sites in chromatin, the regulatory systems that control DNA binding are poorly characterised. Here, we show that NANOG is phosphorylated by casein kinase I, and identify target residues. Phosphomimetic substitutions at phosphorylation sites within the homeodomain (S130 and S131) have site‐specific functional effects. Phosphomimetic substitution of S130 abolishes DNA binding by NANOG and eliminates LIF‐independent self‐renewal. In contrast, phosphomimetic substitution of S131 enhances LIF‐independent self‐renewal, without influencing DNA binding. Modelling the DNA–homeodomain complex explains the disparate effects of these phosphomimetic substitutions. These results indicate how phosphorylation may influence NANOG homeodomain interactions that underpin ESC self‐renewal.

## Graphical abstract

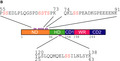

## Abbreviations

**CKI**, casein kinase I

**TF**, transcription factor

The patterns of gene expression during development and cell fate commitment are controlled by a complex web of sequence‐specific transcription factors (TFs). The ability of TFs to regulate gene expression relies on their ability to interact with both DNA and protein partners. Regulation of DNA and partner protein binding, together with modulation of expression levels, enables both temporal control and spatial control of gene expression within a developing organism. One of the best understood mechanisms for regulating TF function is *via* covalent modification of the protein. These modifications include phosphorylation [1], O‐GlcNAcylation [2], acetylation [3] and ubiquitination [4]. Examples of regulation of TF function *via* phosphorylation include STAT3, which is translocated into the nucleus upon phosphorylation [5], c‐myb, which is unable to bind to DNA upon phosphorylation of a residue N‐terminal to the DNA‐binding domain [6] and oestrogen receptor A, which is also blocked from DNA binding upon phosphorylation [7].

In embryonic stem cells (ESCs), the network of TFs that underpin self‐renewal is centred on the triumvirate of factors, NANOG, OCT4 and SOX2 that are often referred to as master transcription factors [8, 9, 10, 11, 12, 13]. Amongst these TFs, NANOG has the ability to drive LIF‐independent ESC self‐renewal when overexpressed [8]. In addition to transcriptional interactions between these TFs [14], each has been shown to be modified *in vivo* by covalent modification. For example, various screens have identified phosphorylated residues in NANOG, OCT4 and SOX2 [15, 16, 17, 18] and phosphorylation has been reported to modify NANOG protein stability [18, 19]. Furthermore, OCT4 and SOX2 are regulated by O‐GlcNAcylation [20] and SOX2 has been demonstrated to be regulated by acetylation [21].

To investigate how NANOG acts through partner proteins to deliver function, we determined the NANOG interactome [22]. This identified > 130 partner proteins of NANOG, including two kinases, one of which is casein kinase I (CKI). The recent demonstration that inhibition of CKI increases reprogramming efficiency of epiblast stem cells [23] suggests that CKI‐mediated phosphorylation is an important regulatory mechanism in pluripotent cells. CKI is a widely expressed enzyme with broad specificity implicated in diverse processes including regulation of circadian rhythms, the cell‐cycle, cell adhesion and cytoskeletal structure [24]. CKI also regulates transcription. For example, CKI regulates the activity of the TFs NFAT‐1 and HIF by direct phosphorylation [25, 26], is a critical component of the β‐catenin pathway [27] and regulates p53 activity [28, 29]. At sites unprimed by prior phosphorylation, CKI has a preference for a negative charge at a position −3 to the target site and an isoleucine at +1 (D/E‐x‐x‐S/T‐I) [30, 31]. In NANOG, 17 of the 61 serine/threonine residues are potential CK1 targets since they either have a negative charge or a phosphorylatable residue at position −3. Here, we report that CKI phosphorylates NANOG in vitro, map sites of phosphorylation and assess the functional effects of phosphorylation at novel sites in the homeodomain on NANOG function in ESCs.

## Materials and methods

### Preparation and phosphorylation of recombinant NANOG

Recombinant NANOG was prepared as described [32]. Phosphorylation was performed by incubating 2 µg NANOG with 0.3 U CKI (New England Biolabs, Ipswich, MA, USA, P6030) in the presence of 200 µm ATP in reaction buffer (50 mm Tris pH7.5, 10 mm MgCl_2_, 5 mm DTT, 0.025% NP‐40) at 30 °C for 2 h. The CKI inhibitors triamterene and D4476 were used at final concentrations of 50, 10 and 1 µm. The reaction was stopped by boiling in Laemmli buffer and the samples loaded onto SDS/PAGE.

### Phosphosite mapping by mass spectrometry

NANOG phosphorylated by CKI was resolved by SDS/PAGE and stained with colloidal Coomassie. The specific NANOG band was excised, washed in water and shrunk in acetonitrile. Gel pieces were reswollen in 50 μL 50 mm Tris/HCl pH 8.0 and reshrunk in acetonitrile. Gel pieces were reduced in 50 mm Tris/HCl pH 8.0, 5 mm DTT at 65 °C for 20 min and alkylated in 20 mm iodoacetamide in 50 mm Tris/HCl pH 8.0 at room temperature for 20 min. Gel pieces were shrunk in acetonitrile, reswollen in 50 mm triethylammonium bicarbonate and reshrunk in acetonitrile. Protein was digested from the gel slice using 5 µg·mL^−1^ mass spectrometry grade trypsin in 50 mm triethylammonium bicarbonate overnight at 30 °C. The gel was shrunk by addition of acetonitrile, swollen in 0.1% TFA and peptides extracted twice with acetonitrile. All supernatants were combined and lyophilised in a SpeedVac prior to mass spectrometry analysis.

Mass spectrometric analysis was performed by LC‐MS‐MS using a linear ion trap‐orbitrap hybrid mass spectrometer (Orbitrap‐Classic, Thermo, Waltham, MA, USA) equipped with a nanoelectrospray ion source (Thermo) and coupled to a Proxeon EASY‐nLC system. Peptides (in 0.1% formic acid, 2% acetonitrile) were injected *via* a 2 cm trap column (Nano Separations, NS‐MP‐10 BioSphere C18, 5 μm, 120 Å, 360/100 μm) onto a Thermo (Part No. 160321) Acclaim PepMap100 reverse phase C_18_ 3 μm column (75 μm × 15 cm) with a flow of 300 nL·min^−1^ and eluted with a 45 min linear gradient of 95% solvent A (2% acetonitrile, 0.1% formic acid in H_2_O) to 40% solvent B (90% acetonitrile, 0.08% formic acid in H_2_O), followed by a rise to 80% B at 48 min. The instrument was operated with the 'lock mass' option to improve the mass accuracy of precursor ions, and data were acquired in the data‐dependent mode, automatically switching between MS and MS‐MS acquisition. Full scan spectra (*m*/*z* 340–1800) were acquired in the orbitrap with resolution *R* = 60 000 at *m*/*z* 400 (after accumulation to an FTMS Full AGC Target; 1 000 000; MSn AGC Target; 100 000). The 5 most intense ions, above a specified minimum signal threshold (20 000), based upon a low‐resolution (*R* = 15 000) preview of the survey scan, were fragmented by collision‐induced dissociation and recorded in the linear ion trap (Full AGC Target; 30 000. MSn AGC Target; 5000). Multistage activation was used to provide a pseudo‐MS3 scan of any parent ions showing a neutral loss of 48.9885, 32.6570, 24.4942, allowing for 2+, 3+ and 4+ ions, respectively. The resulting pseudo‐MS3 scan was automatically combined with the relevant MS2 scan prior to data analysis.

RAW files from the OrbiTrap‐Classic were analysed by Proteome Discoverer 1.4‐SP1 (Thermo) using the phosphoRS [33] node and searched using mascot 2.4.1 (www.matrixscience.com) against an in‐house database that contained the NANOG Q80Z64.1 sequence. Searches allowed for variable modifications; dioxidation (M), oxidation (M), phospho (ST), phospho (Y) and a fixed modification: carbamidomethyl (C). Peptide mass tolerance was ± 10 ppm, and MS2 fragment mass tolerance was ± 0.6 Da.

### Cell transfections and self‐renewal assays

For episomal assessment of function of NANOG mutants, DNA was transfected into E14/T cells using Lipofectamine 2000 (Thermo Fisher, 11668027). One million cells were plated per well of a 6‐well plate in 2.0 mL Gmemß/10% FCS, LIF 30–60 min before DNA addition. 3 μg DNA was diluted into 250 μL of serum‐free Gmemβ for each transfection. Separately, 3 μL of Lipofectamine 2000 was diluted into 250 μL of serum‐free Gmemβ and incubated at room temperature for 5 min before mixing with the DNA solution and incubation at room temperature for 20 min. The transfection cocktail was then added to the cells and the plates rocked gently before being placed in the incubator. Cells were trypsinised 24 h later, plated at 5 × 10^4^ cells per 9‐cm dish in medium supplemented with 2 μg·mL^−1^ puromycin and medium replaced every 2 days. Colonies were photographed after 12 days following fixation and alkaline phosphatase staining using a leucocyte alkaline phosphatase kit (Sigma kit 86‐R, St. Louis, MO, USA) [34].

For generation of stable lines, plasmids encoding NANOG and NANOG mutants were linearised with ScaI and introduced into 44Cre6 NANOG null cells [35] *via* electroporation. After selection in media containing puromycin, 600 cells were plated in triplicate in 6‐well plates plus and minus LIF. Plates were stained for alkaline phosphatase as described above. Antibodies used for determining expression levels are anti‐NANOG, A300‐397A, Bethyl Laboratories and anti‐LAMIN B1, Ab16048, Abcam (Cambridge, MA, USA).

### Electrophoretic mobility shift assay

All assays used oligonucleotide labelled at the 5′ end of the upper strand with IR700 dye. The DNA probe corresponds to the NANOG‐binding site in Tcf3 promoter CTGTTAATGGGAGC [36]. Protein was added to oligonucleotide and buffer to give final concentrations of 10 mm HEPES pH7.9, 10% glycerol, 50 mm KCl, 10 mm NaCl, 0.4 mm EDTA, 2.5 mm DTT, 0.25% Tween 20, 100 mg·mL^−1^ dI:dc, 1 mg·mL^−1^ BSA, 10 nm oligonucleotide, 250 nm protein. Reactions were incubated at room temperature for 30 min before analysis on 5% acrylamide gels [37]. Data were collected on a LICOR Odyssey Fc. Recombinant His‐tagged homeodomains were expressed in *E. coli* with induction of protein expression at 30 °C for 3 h. Bacterial lysates were prepared in 25 mm Tris pH8.0, 500 mm NaCl, 30 mm imidazole and loaded onto His‐select resin (Sigma, P6611). After washing in the same buffer, protein was eluted in 25 mm Tris pH8.0, 500 mm NaCl, 250 mm imidazole. Proteins were dialysed against 25 mm Tris pH8.0, 500 mm NaCl and used directly in EMSAs.

### Mutagenesis

Synthetic DNAs (IDT) encoding the mutations were inserted into pPyCAG‐NANOG [8] cut with PpuMI and NotI.

### Chromatin immunoprecipitation

Ten million E14Tg2a cells were resuspended in 3 mL GMEM/FCS (10%) and cross‐linked with 1% formaldehyde (Sigma, P6611) for 10 min at room temperature. The reaction was quenched with 0.125 mm glycine for 5 min at room temperature. Cells were pelleted and washed twice with cold PBS. Cells were resuspended in 300 μL of swelling buffer (5 mm Pipes pH 8, 85 mm KCl) supplemented with 1× protease inhibitor cocktail (Roche) and 0.5% NP‐40. The suspension was incubated for 20 min on ice. Nuclei were pelleted and resuspended in 1.5 mL of 0.1% SDS, 1% Triton X‐100, 2 mm EDTA, 20 mm Tris/HCl pH8, 150 mm NaCl, supplemented with 1× protease inhibitor cocktail. Samples were sonicated at 4 °C in a Bioruptor (Diagenode). 20 μg of sonicated chromatin was used per ChIP. Immunoprecipitation with 5 µg rabbit anti‐NANOG [32] was performed overnight with rotation at 4 °C, in a final volume of 1 mL. Immunocomplexes were purified using Protein‐G Dynabeads (Lifetech, Waltham, MA, USA). Washing and elution were as described in Ref. [38]. The sequences of the oligos used for ChIP‐Q‐PCR are as follows: Xin1: AACCCTTTTAAGTCCACTGTAAATTCC and TAGAGAGCCAGACAATGCTAAGCC; Xex1b: CATCAGGCTTGGCAGCAA and TTCATCAGCAATGTCATATCAAACA; EE2: TGCTGGTGGTATTCAACTGC and CGGATCTGTGGAATTCGTG; E7: TAAGGGACACCTCCCTAGCC and CCATACCCCACACACCATGT; Otx2 A: CAAGAACAAAACCCCACGCC and GTGCCAGCCAATGAGTCCTA; Otx2 B: GTGGAAGGGCGTCTAGAAGG and GCAGTCAATGGGCTGAGTCT [39].

### Modelling a mouse NANOG homeodomain–DNA structure

NANOG homeodomain sequences from mouse and human were aligned with ClustalOmega [40]. The coordinates of protein chain A from the mouse NANOG homeodomain crystal structure (PDBID: 2VI6) were renumbered and superimposed on chain A of the human NANOG HD cocrystal structure with DNA (PDBID: 4RBO) with SUPERPOSE in the CCP4 software suite [41]. The side chains of serines 130 and 131 were phosphorylated, and the structures were visualised with pymol (Schrodinger Inc, New York, NY, USA).

## Results

### NANOG is phosphorylated by casein kinase I at multiple sites

**Fig. 1:**
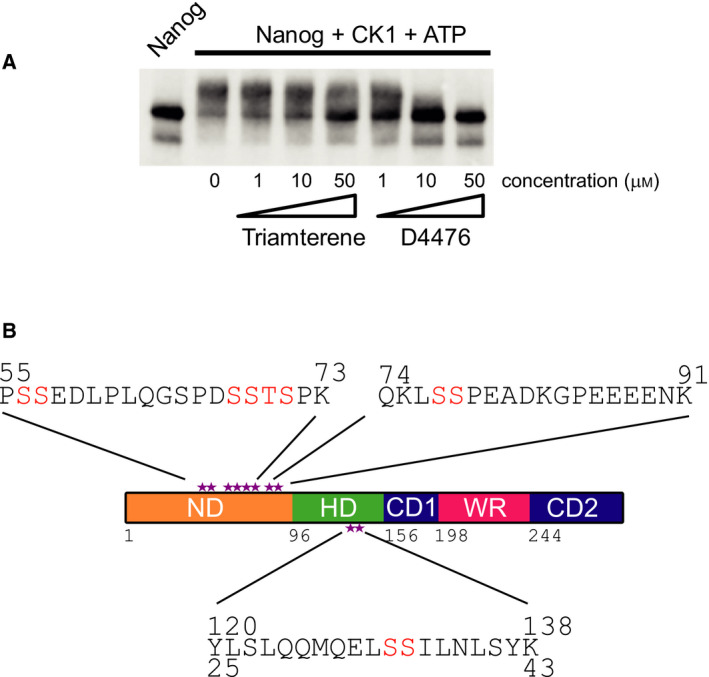
NANOG is phosphorylated by casein kinase I. (A) Immunoblot of recombinant NANOG incubated with CKI and ATP in 0, 1, 10 or 50 µmtriamterene or D4476. (B) Domain diagram of NANOG with phosphorylation sites identified by mass spectrometry marked as stars. ND – N‐terminal domain, HD – homeodomain, CD1 – C‐terminal domain 1, WR – tryptophan repeat, CD2 – C‐terminal domain 2. Sequences of phosphorylated NANOG peptides are shown with potential phosphorylation sites highlighted. For all peptides, NANOG residue numbering is shown above the sequence. For the homeodomain‐derived peptide, the canonical homeodomain numbering is shown below the sequence.

The presence of CKI in the NANOG interactome [22] combined with the potential of kinases to modulate the function of TFs [5, 6, 7] led us to investigate whether CKI could modulate NANOG function by phosphorylation. Recombinant NANOG was therefore incubated with recombinant active CKI and ATP *in vitro*. This resulted in a clear shift in molecular weight of NANOG, which can be indicative of one or more phosphorylation events (Fig. 1A). The shift in mobility was inhibited by inclusion of either of the CKI inhibitors triamterene or D4476, suggesting that retarded electrophoretic mobility is indeed a result of NANOG phosphorylation by CKI. The greater degree of inhibition by D4476 is consistent with the lower IC50 of CKI inhibition by D4476 (0.3 µm) compared to triamterene (6.9 µm) [42, 43].

To identify phosphorylation sites, NANOG was incubated with CKI and ATP, subjected to SDS/PAGE and the stained bands analysed by mass spectrometry. Three phosphorylated peptides were detected (Fig. 1B and Table 1), two in the N‐terminal domain and one in the homeodomain. The most N‐terminal phosphorylated peptide was detected in singly and doubly phosphorylated forms. The single phosphorylation is on either serine 56 or 57, with the mass spectrometry data indicating an equal probability of phosphorylation on each residue. The doubly phosphorylated form is also phosphorylated on either serine 56 or 57. In addition, the doubly phosphorylated form is likely to be modified on either serine 68 or 69, although there is also a low probability that this second phosphorylation could occur on either threonine 70 or even serine 71 (Table 1). The second phosphorylated N‐terminal peptide contains a single modification that is likely to be on serine 77, although the mass spectrometry data indicate a 10% probability that serine 78 could be phosphorylated. These results are consistent with previous work showing that NANOG expressed in 293T cells is phosphorylated on either serine 56 or 57 and either serine 77 or 78 [19]. The third phosphopeptide is derived from the homeodomain. The mass spectrometry data suggest that the modified residue is likely to be serine 131. Notably, S131 is a good match to the consensus CKI sequence (D/E‐x‐x‐S/T‐I), since residue 128 is a glutamate and residue 132 is an isoleucine [30, 31]. However, the possibility that serine 130 is phosphorylated cannot be discounted. S130 and S131 correspond to residues 35 and 36 in the canonical homeodomain numbering system [44]. Therefore, the phosphorylation sites identified here by mass spectrometry include residues previously reported as phosphorylation sites [19, 45, 46] as well as novel sites of phosphorylation within the homeodomain; these are highlighted on a domain diagram of NANOG (Fig. 1B).

**Table 1:** Identification of NANOG sites phosphorylated by CK1 using mass spectrometry.

Peptide sequence^a^	Phos sites	M/Z (Da)	Ion score	phosphoRS probabilities^33^
55‐PSSEDLPLQGSPDSSTSPK‐73	1	1004.9417	59	S56:50.0 S57:50.0
55‐PSSEDLPLQGSPDSSTSPK‐73	2	1044.9257	38	S56:50.0 S57:50.0 S68:47.5 S69:47.5 T70:4.5 S71:0.5
74‐QKLSSPEADKGPEEEENK‐91	1	698.9794	39	S77:90.0 S78:10.0
120‐YLSLQQMQELSSILNLSYK‐138	1	780.0541	30	S130:8.9 S131:91.1

^a^ Sequences are numbered according to the primary NANOG sequence.

### Mutagenesis of phosphorylation sites within the NANOG homeodomain

**Fig. 2:**
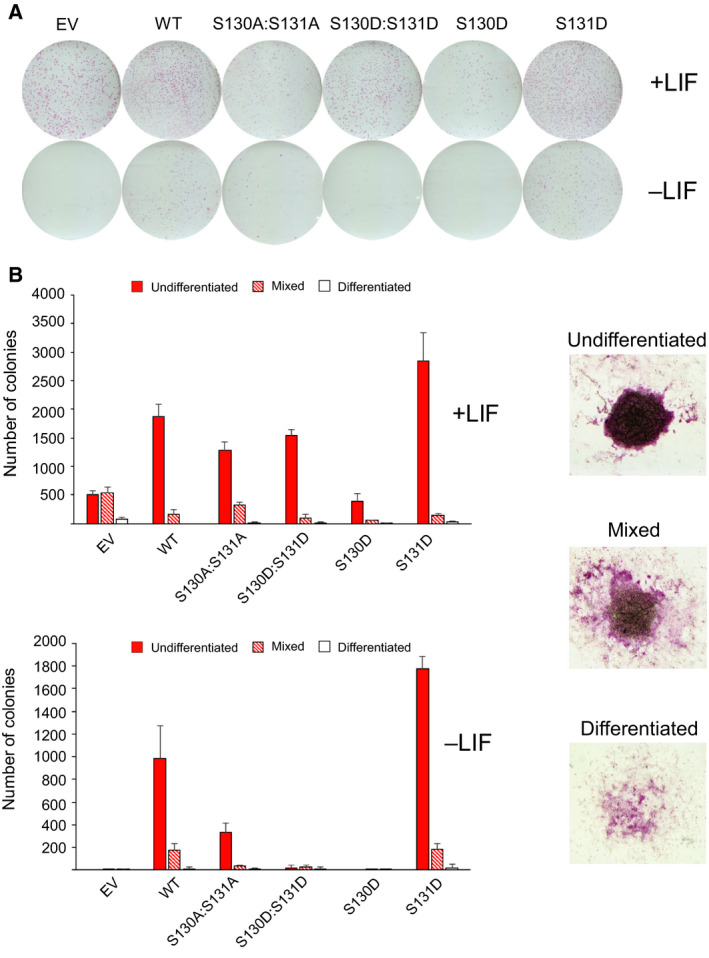
Self‐renewal assays of E14/T cells transfected with NANOG variants. (A) E14/T ESCs were transfected with pPyCAGIP plasmids encoding the indicated NANOG mutants and cultured in media containing puromycin in the presence or absence of LIF (EV, empty vector; WT, wild‐type NANOG). After 12 days, plates were stained for alkaline phosphatase activity. Assays were performed in triplicate. (B) Left, Quantitative assessment of colony morphologies from (A) determined in the presence or absence of LIF. Data shown are from a single experiment (data and error bars are the means and standard deviations, respectively, from three technical replicates) and are representative of three independent biological replicates. Biological replicates are shown in Fig.S1. Right, examples of colony morphologies containing undifferentiated ES cells, differentiated cells or a mix of both.

The identification of CKI‐mediated phosphorylation within the NANOG DNA‐binding domain suggests that these modifications may regulate NANOG function. To test for possible roles of phosphorylation, the two potential phosphorylation sites within the homeodomain were mutated simultaneously, either to alanine or to the phosphomimetic residue aspartate. In addition, both residues were individually mutated to aspartate. To determine whether these mutations affected the defining capacity of NANOG to confer LIF‐independent self‐renewal on transfected ESCs [8], these mutants were cloned into constitutive episomal expression plasmids and transfected into E14/T ESCs to rapidly assess ESC self‐renewal (Fig. 2A,B). Examples of colony morphologies containing undifferentiated ES cells, differentiated cells or a mix of both are shown in Fig. 2B. Mutagenesis of both residues to alanine has little effect on the ability of NANOG to confer LIF‐independent self‐renewal. However, mutagenesis to aspartate either singly or doubly has a marked effect. Substitution of S130 by aspartate (S130D) abolishes the LIF‐independent self‐renewal function of NANOG. Moreover, in the presence of LIF, S130D acts as a dominant interfering mutant since self‐renewal is decreased below the level obtained by transfection of an empty vector control. In contrast, a phosphomimetic at position 131 increases the number of self‐renewing colonies formed in either the presence or absence of LIF (Fig. 2B and Fig. S1A,B).

**Fig. 3:**
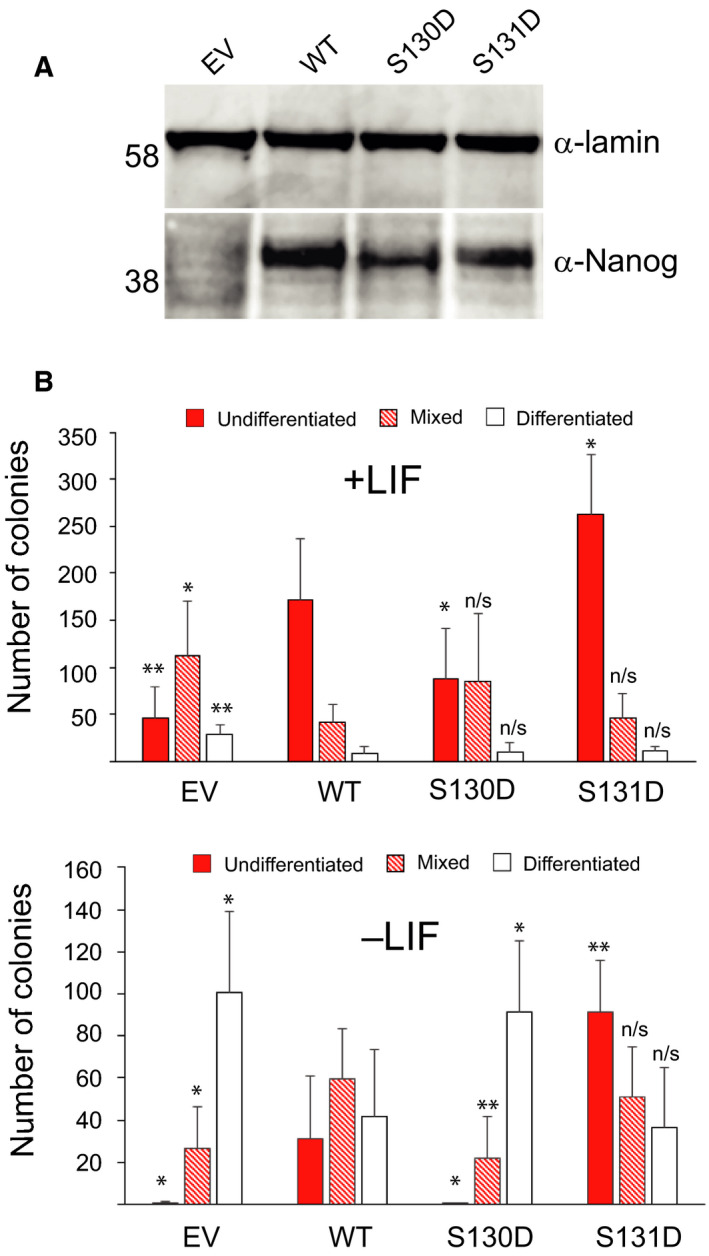
Self‐renewal assays of*NANOG*null ESCs rescued with NANOG variants. (A)*NANOG* ^−/−^ESCs (44cre6) were electroporated with linearised pPyCAGIP plasmids encoding the indicated NANOG mutants and selected in puromycin LIF (EV, empty vector; WT, wild‐type NANOG). After expansion, cell extracts were assayed for expression level by immunoblotting. (B) Quantitative assessment of colony morphologies of stably expressing lines from (A) plated at clonal density in the presence or absence of LIF and stained for alkaline phosphatase activity after 6 days. Data and error bars are the means and standard deviations, respectively, of three independent experiments. Significance of variation from WT *P < 0.1, **P < 0.05 and n/s – not significant (Student’s*t*‐test).

To attempt to verify the findings from E14/T ESCs, and to further investigate the effect of mutating serine 130 and 131, stably expressing ESC lines were generated in a *Nanog* ^−/−^ background [35] and the ability of the mutants to confer LIF‐independent self‐renewal assayed (Fig. 3). Immunoblot analysis indicated that the stable cells express comparable levels of NANOG (Fig. 3A). Consistent with the clonal self‐renewal results from E14/T cells, S131D increases self‐renewal efficiency in the presence and absence of LIF whereas S130D decreases self‐renewal efficiency (Fig. 3B). Notably, the dominant‐negative effect of S130D observed in E14/T ESCs is not observed in a *Nanog* ^−/−^ background, consistent with the mutant protein interfering with WT NANOG function. These data suggest that individual phosphorylation at the adjacent sites 130 and 131 has opposite effects on the biological activity of NANOG. Mimicking phosphorylation at 130 abrogates NANOG function, and in wild‐type cells interferes with the function of endogenous NANOG. In contrast, mimicking phosphorylation at 131 enhances both the total number and the proportion of cells that undergo efficient self‐renewal.

### DNA‐binding properties of mutants

**Fig. 4:**
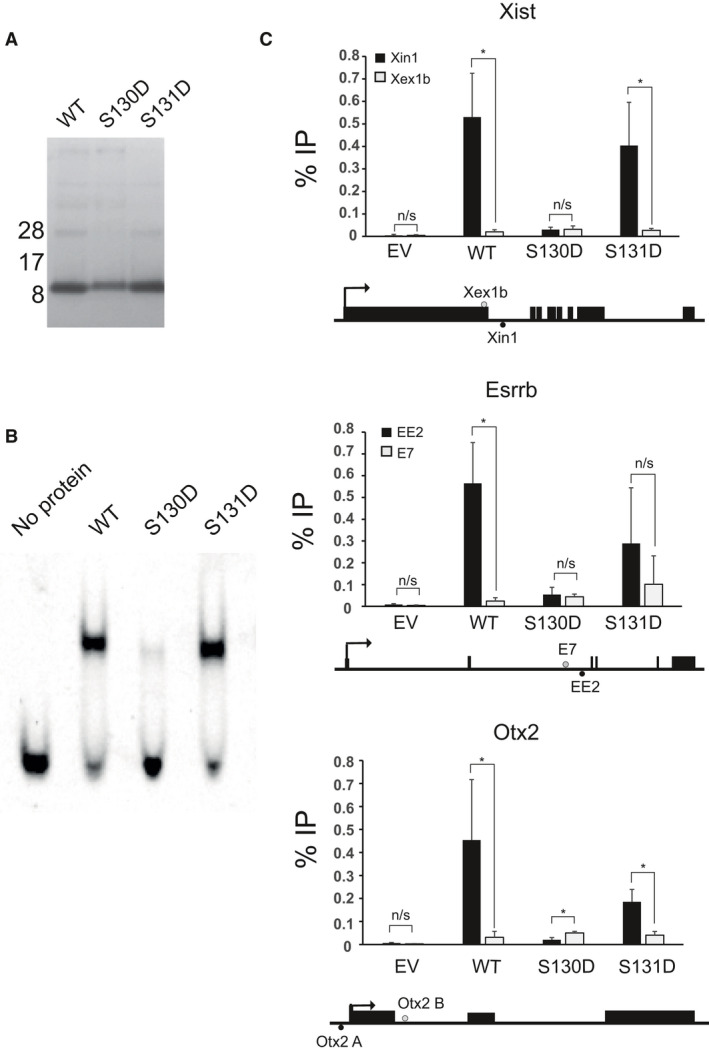
DNA binding by NANOG mutants. (A) SDS/PAGE of purified homeodomains expressed in*E. coli*.(B) EMSAs with recombinant homeodomains performed using a probe corresponding to the NANOG‐binding site within the*Tcf3*locus [36]. (C) ChIP‐PCR of NANOG at known binding sites. ChIP was performed with NANOG antibody using the stable populations described in Fig. 3. Three known NANOG‐binding sites within*Xist*,*Esrrb*and*Otx2*were assessed; regions within the same genes which do not bind NANOG provided negative controls. The positive binding regions (Xin1, EE2 and Otx2 A) are diagrammed below each gene and negative control regions (Xex1b, E7 and Otx2 B) above each gene. Data are mean of three replicates. Error bars are standard deviation. *P < 0.05, n/s – not significant.

As S130 and S131 are in the NANOG homeodomain, their phosphorylation may alter the ability of NANOG to bind DNA. To compare the abilities of NANOG mutants to bind DNA, recombinant homeodomains encompassing the D > A mutations were purified for use in electrophoretic mobility shift assays (EMSAs; Fig. 4A). While the S131D homeodomain has a DNA‐binding capacity similar to that of the wild‐type NANOG homeodomain, EMSAs indicate that the DNA‐binding capacity of S130D homeodomain is severely impaired (Fig. 4B).

To investigate the ability of NANOG mutants to bind to chromatin, ChIP was performed using the stable lines described above. Mutation of S131 to aspartate has little effect on the ability of NANOG to bind chromatin (Fig. 4C). However, S130D was not localised to chromatin at any of the three loci assayed. This is in agreement with the reduced capacity of S130D to bind DNA *in vitro* (Fig. 4B). Together these data indicate that while phosphorylation of NANOG on S131 has no effect on DNA binding, phosphorylation of S130 may disrupt the binding of NANOG to target sites in chromatin.

### Modelling of the NANOG homeodomain–DNA complex

**Fig. 5:**
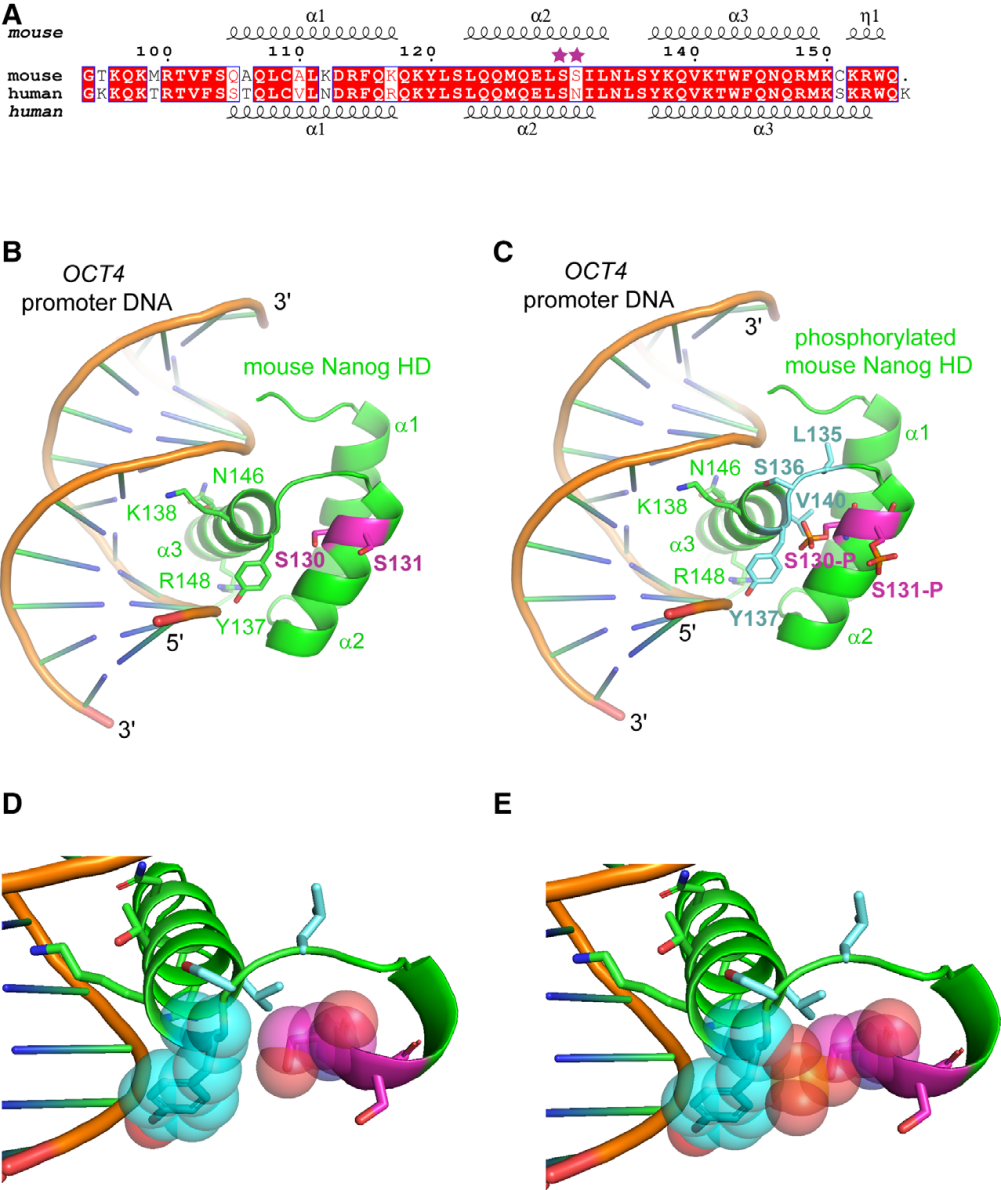
Model of mouse NANOG homeodomain in complex with DNA. (A) Alignment of the homeodomain (HD) sequences of mouse (amino acids 94–155) and human NANOG (93–155). White letters on red background indicate conserved amino acids; red letters on white background indicate similar amino acids. Two potential phosphorylation sites, S130 and S131, are highlighted with stars. Secondary structure elements of the mouse NANOG HD crystal structure (PDBID:2VI6) and the human NANOG HD cocrystal structure with DNA (PDBID:4RBO) are shown above and below the alignment, respectively. (B) Structural model of mouse NANOG HD bound to*OCT4*promoter DNA, based on the human NANOG HD:DNA cocrystal structure. Four amino acids in α‐helix 3 that contact DNA (Y137, K138, N146, R148) are shown as sticks, along with S130 and S131 (purple). (C) Structural model of mouse NANOG HD, phosphorylated at S130 (S130‐P) and S131 (S131‐P), bound to*OCT4*promoter DNA. Phosphorylation of S130 introduces steric clashes with the amino acid residues shown in blue (L135, S136, Y137, V140) sticks. (D, E) Expanded view of region around S130. Atoms of Y137 and Ser130/ pSer130 are shown as spheres representing van der Waal radii. Residues in blue as per C.

The differing effects observed between mutating S130 and S131 on DNA and chromatin binding are intriguing, given that the residues are adjacent in the primary NANOG sequence. While no three‐dimensional structure is available for mouse NANOG in complex with DNA, the tertiary structure of the mouse homeodomain has been solved [36], as has the structure of the human homeodomain in complex with DNA [47]. An alignment of the mouse and human NANOG homeodomains (Fig. 5A) was used to construct a three‐dimensional model of mouse NANOG in complex with DNA (Fig. 5B). The root mean square deviation of superposed mouse and human NANOG HD chains was 0.91 Å over all atoms. Highlighted on the model are S130 and S131, which are present at the C‐terminal end of homeodomain α‐helix 2. This shows that neither S130 nor S131 are in direct contact with DNA (Fig. 5B). A three‐dimensional model of mouse NANOG in complex with DNA was also prepared after addition of a phosphate group to either S130 or S131 (Fig. 5C). Mutation of S130 to aspartate creates steric clashes with Tyr 137 and Val 140 at the N terminus of α‐helix 3 (or in an alternative rotamer conformation with backbone atoms of Leu 135 and Ser 136; Fig. 5D,E). The phosphorylation of Ser 130 is therefore likely to lead to a change in the position or orientation of at least the N‐terminal end of α‐helix 3, which may interfere with DNA binding. In particular, the critical contacts made with the DNA backbone by Tyr 137 at the N‐terminal end of this helix are likely to be disrupted. In contrast to S130, the side chain of S131 faces away from helix 3, making it unlikely that phosphorylation of this residue would directly influence DNA binding. This is consistent with the EMSA and ChIP results showing that S131D retains the ability to bind DNA and to localise to NANOG target sites in chromatin (Fig. 4B,C).

## Discussion

The NANOG interactome contains more than 130 proteins of which only two are kinases (casein kinases I and II) [22]. A role for CKI in pluripotent cells was recently reported, with the demonstration that CKI inhibition improved the efficiency of reprogramming of epiblast stem cells to ESCs [23, 42]. To further investigate CKI function in ESCs, the ability of CKI to directly phosphorylate NANOG, one of the central members of the pluripotency gene regulatory network, was investigated. This showed that NANOG is a CKI substrate in vitro. Of the sites we identify as phosphorylated by CKI, residues 70 and 131 are previously unreported phosphorylation sites that each have a negatively charged residue at position −3 that fits the D/E‐x‐x‐S/T consensus for CKI phosphorylation [30, 31]. Of the remaining sites, residues 68 and 71 have serines at the −3 position which could act as priming sites in vivo. Consistent with this possibility, serine 65 has been reported to be phosphorylated in cells [19, 45]. The probability of serine 71 being phosphorylated by CKI is low (Table 1), and furthermore, there has been no in vivo identification of a potential priming phosphorylation on serine 68. We also identified additional CKI sites that have been reported as sites of phosphorylation. Specifically, phosphorylation of mouse NANOG at S56/S57 has been demonstrated in ESCs and in 293T cells [19, 45]. In addition, protein kinase A can phosphorylate recombinant human NANOG on the residue equivalent to S78 in mouse NANOG [46]. However, these latter sites do not match the consensus for CKI phosphorylation. Moreover, functional roles have not been assigned to any of the above phosphorylated residues. Interestingly, previous studies have not reported phosphorylation within the NANOG homeodomain. Our work identifies a novel phosphorylation site(s) within the homeodomain which could potentially occur on S130 and/or S131 (corresponding to residues 35 and 36 in the canonical homeodomain numbering system [44]).

Intriguingly, phosphorylation of residues within other homeodomains has functional consequences. For example, phosphorylation of PRH at a serine immediately before the start of helix 3 of the homeodomain (residue 41 of the HD numbering scheme) decreases DNA binding [48]. Structural modelling suggests this is due to a disruption of the side chain interactions that mediate DNA binding [49]. This is interesting relative to our study, since the phosphate group added to residue 41 of the PRH HD is in a 3D space close to that occupied by introduction of a phosphate group to homeodomain residue 35 (S130) of NANOG. Of further note, mimicking phosphorylation of NANOG HD residue 35 reduces DNA binding and abolishes the biological activity of NANOG, echoing the effect of mimicking phosphorylation at PRH HD residue 41 on abolition of activity *in vivo* [48]. In contrast, phosphorylation within homeodomains can also increase DNA affinity. This is the case for Nkx2.5, where phosphorylation of a residue between the first and second helices of the homeodomain increases affinity for DNA and is also important for transcriptional activity [50]. Phosphorylation of homeodomains can also have functional consequences without affecting DNA binding. For example, Ftz can be phosphorylated by protein kinase A on a threonine in the first helix of the homeodomain. Substitution of threonine with alanine does not affect DNA binding but fails to rescue the segmentation defect in *ftz* null drosophila embryos [51].

Mass spectrometry identified a singly phosphorylated residue in the NANOG peptide covering serines 130 and 131. While it is possible that NANOG could either be phosphorylated on 130 or 131, probability analysis of the mass spectrometry data suggests that phosphorylation of residue 131 is the more likely. Moreover, it is notable that phosphorylation at S131 is consistent with studies of CKI specificity, since the residue at −3 is a glutamate and the residue at +1 is an isoleucine [30, 31]. Nevertheless, to obtain comprehensive insight into the potential functional consequences of phosphorylation of either of these residues, we mimicked phosphorylation by mutagenesis of serines to aspartate. Intriguingly, aspartate substitution of either S130 or S131 of NANOG had functionally distinct effects. Similar to the effect of phosphorylation of S41 within the PRH homeodomain [48], aspartate substitution of S130 reduces the capacity of NANOG to bind DNA. Consistent with this loss of DNA‐binding activity, S130D showed a loss of ESC self‐renewal capacity. Moreover, the modelled structure of a NANOG–DNA complex shows that phosphorylation of S130 would cause steric hindrance to the spatial disposition of residues L135, S136, Y137 and V140 (HD residues 40, 41, 42 and 45). Notably, this would include interference with the critical contacts between the DNA backbone and Y137. Alanine substitution of Y137 decreases DNA binding activity [47] and impairs the reprogramming activity of mouse NANOG [52]. These contact disruptions differ in detail to those seen in PRH, where phosphorylation of S41 disrupts a hydrogen bonding network centred on Q44.

In contrast, aspartate substitution of S131 increased the ESC self‐renewal capacity of NANOG without affecting DNA binding. The results from self‐renewal assays demonstrate that a phosphomimetic at position 131 has a large effect on NANOG function, increasing its ability to support LIF‐independent self‐renewal several fold. It will be interesting in future to see whether S131D also enhances reprogramming to an ES cell state [52, 53, 54]. In the model of the NANOG–DNA complex, the side chain of serine 131 points away from the DNA. This is consistent with the lack of an effect on DNA binding for S131D as assayed by EMSA. Therefore, instead of mediating protein–DNA interactions S131 may mediate interaction with NANOG partner proteins. Although NANOG is known to interact with > 130 partner proteins [22, 55, 56], the residues within NANOG that mediate these interactions have been identified in only a minority of cases [22, 57]. The fact that modification of S131 results in an increase in activity that is independent of DNA binding suggests that reversible modification of the NANOG homeodomain may be used as a switch to regulate interactions between NANOG and partner proteins and to thereby modulate ESC self‐renewal efficiency. Future work should address this issue and the alternative possibility that S131D affects protein stability as is the case for the SOX17 gain‐of‐function mutant SOX17 V118M [58].

## Author contributions

IC and NPM conceived the project, designed the experiments and wrote the manuscript. NPM performed phosphorylation assays, constructed mutants and performed EMSAs and ChIP assays. GMF and JV performed phosphorylation analysis. JMR modelled the NANOG–DNA structure. DC performed cell culture.

## Supporting information


**Fig S1.** Replicate self‐renewal assays of E14/T cells transfected with NANOG variants.
