# Supplementary material for: Phosphorylation of NANOG by casein kinase I regulates embryonic stem cell self‐renewal
Source: FEBS Lett. 2020 Nov 18;595(1):14–25. doi: 10.1002/1873-3468.13969 (PMC7839479; doi:10.1002/1873-3468.13969)
Supplement: Supplementary file 1 — Fig S1. Replicate self‐renewal assays of E14/T cells transfected with NANOG variants. [file FEB2-595-14-s001.pdf]

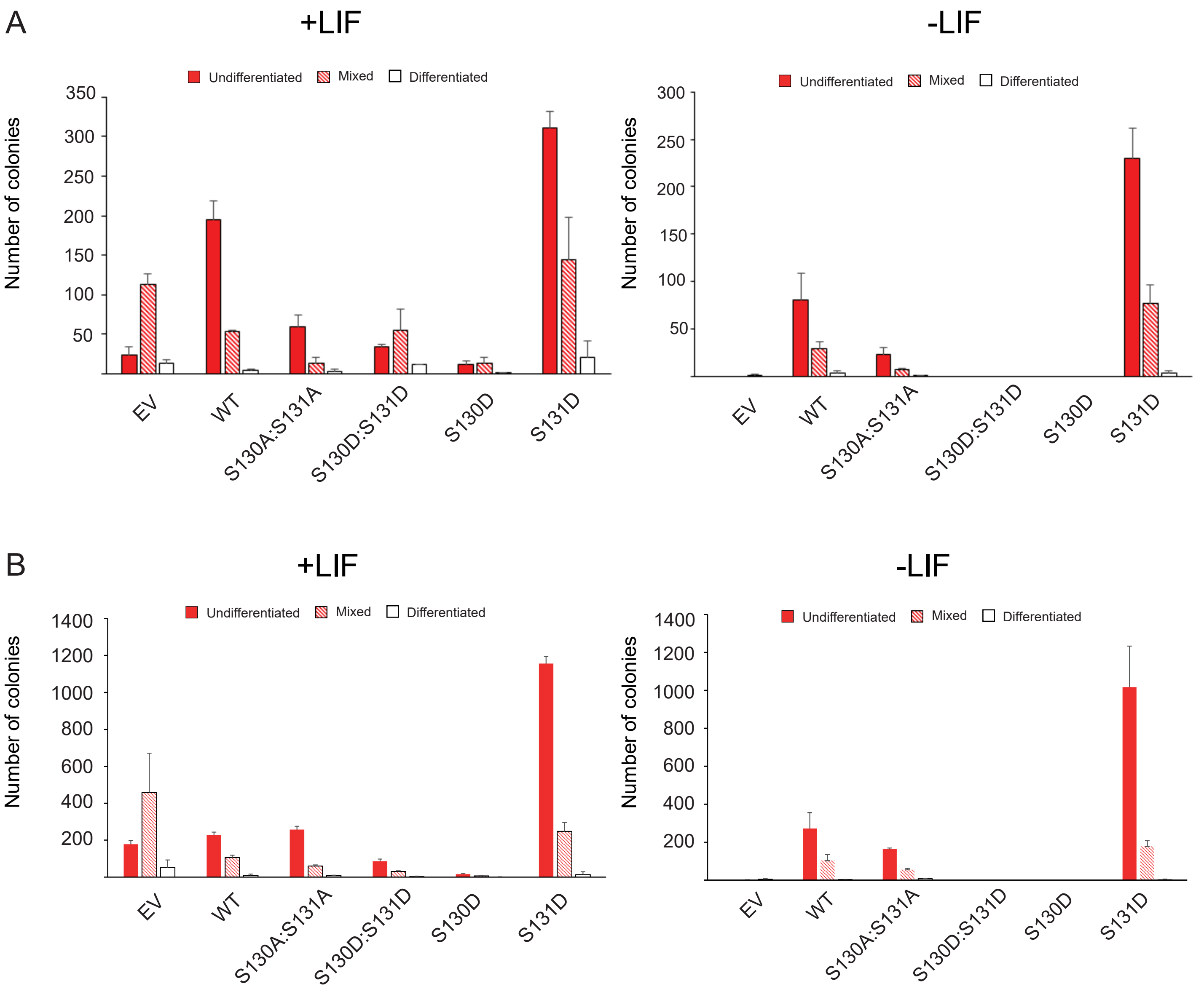

Supplementary Figure 1. Replicate self-renewal assays of E14/T cells transfected with NANOG variants. A. and B. Assays are replicates of experiment described in Fig. 2.
